# Factors associated with urinary tract infection in the early phase after performing intermittent catheterization in individuals with spinal cord injury: a retrospective study

**DOI:** 10.3389/fmed.2023.1257523

**Published:** 2023-11-17

**Authors:** Huayi Xing, Hongyue Dai, Baohua Li, Xiaoning Yuan, Xiaoxuan Liu, Guoqing Cui, Nan Liu, Fin Biering-Sørensen

**Affiliations:** ^1^Department of Rehabilitation Medicine, Peking University Third Hospital, Beijing, China; ^2^Department of Nursing, Peking University Third Hospital, Beijing, China; ^3^Department of Hospital-Acquired Infection Control, Peking University Third Hospital, Beijing, China; ^4^Clinic for Spinal Cord Injuries, Rigshospitalet, University of Copenhagen, Copenhagen, Denmark

**Keywords:** spinal cord diseases, urinary tract infections, intermittent catheterization, neurogenic bladder, neurorehabilitation

## Abstract

**Objectives:**

To investigate the occurrence rate of urinary tract infections (UTIs) in the early phase after performing intermittent catheterization (IC) and to explore the possible factors associated with UTIs after performing IC among people with spinal cord injury (SCI).

**Setting:**

An inpatient rehabilitation department of a teaching hospital in China.

**Design:**

Retrospective chart review.

**Methods:**

A retrospective chart review was carried out for traumatic and non-traumatic SCI patients after performing IC during their inpatient stay. Demographic information, comorbidity of diabetes, urine analysis results before IC, method of IC (sterile or clean), use of bladder irrigation, cessation of IC and its reasons, and UTI events were collected.

**Results:**

A total of 183 adult individuals were included, of which 60 (32.8%) of them were women. The median age was 49.0 years. The median time post-injury was 2 months. The overall occurrence rate of UTI after performing IC was 1.31 (95% confidence intervals: 0.96–1.77) events per 100 days. Sixty-nine (37.7%) patients discontinued IC during hospitalization, and UTIs were the leading reason for cessation (50.7%). Female sex, use of antibiotics for infections other than UTI, and use of bladder irrigation were found to be associated with a lower occurrence rate of UTI in the early phase after performing IC, with an odds ratio of 0.38 (*p* = 0.019), 0.20 (*p* = 0.022), and 0.24 (*p* < 0.001), respectively.

**Conclusion:**

UTI after performing IC is prevalent among people with SCI. The study indicated that antibiotic prophylaxis and routine bladder irrigation might be associated with the reduction in UTI in the early phase after performing IC. Further research is needed to provide more evidence.

## Introduction

Urinary tract infection (UTI) is a common complication among people with spinal cord injury (SCI) (1). It has been reported that UTIs occur at an overall incidence rate (IR) of 0.68–2.5 events per SCI patient per year (2, 3). UTIs are related to impaired lower urinary tract function and/or inappropriate bladder management (such as prolonged indwelling catheterization and voiding with Valsalva maneuver) after SCI. They may cause extended length of stay, delayed rehabilitation programs, and impaired quality of life (4). Furthermore, serious consequences of UTIs such as renal failure and septicemia are associated with declined life expectancy in individuals with SCI (5, 6).

The incidence of UTIs after SCI is related to the method of urine drainage (7, 8). Indwelling urethral catheterization (IDC) is most often used in the early phase after an injury, but those with indwelling urethral catheters have a high risk of developing UTIs (1). Intermittent catheterization (IC) is recommended in several clinical guidelines and should be initiated as soon as possible (9–12). Compared to IDC, IC is associated with lower rates of UTI (13). Furthermore, the maintenance of bladder volume and regular emptying intervals appear to be protective for renal function (14). Thus, IC is widely accepted as the preferred method of bladder management for individuals with SCI and neurogenic bladder dysfunction. Sterile IC (*SIC*) is usually performed by clinical staff and clean IC (CIC) by the patients themselves or by their caregivers.

UTI is one of the most common complications after performing IC among chronic SCI populations (>1 year post-injury) (14). Recurrent UTIs are the major reasons for CIC cessation in individuals with SCI in a registry research (15). Another study using mixed bladder management techniques (IDC, CIC, suprapubic indwelling catheter, and reflex voiding) reported that the IR of UTI was 6.84 per 1,000 days in individuals with acute SCI performing CIC (7), and the authors found patients suffering from UTI after IC in clinical practice; however, factors associated with the occurrence of UTI, especially in the early phase after switching to IC in SCI individuals, were not investigated.

As UTIs after performing IC are quite prevalent, the authors need to find out their associated factors during this time window for better treatment and prevention strategies. However, few empirical studies have explored those factors among people with SCI. In this study, the authors investigated the events of UTI in the early phase after performing IC during an inpatient stay; the study aimed to (1) estimate the occurrence rate of UTIs after performing IC and (2) explore the factors associated with UTIs in the early phase after performing IC.

## Methods

### Participants

A retrospective chart review was performed for traumatic and non-traumatic SCI admissions to the Department of Rehabilitation Medicine, Peking University Third Hospital, Beijing, China, from January 2015 to December 2019. The study protocol was approved by the Clinical Research Ethics Board of Peking University Third Hospital (LM2020271). Participants were identified through diagnostic codes of “spinal cord injury” or “tetraplegia”/“paraplegia.” The inclusion criteria were age > 18 years and IC being initiated during individuals’ inpatient stay. Individuals using other methods for bladder management were excluded.

### Intermittent catheterization protocol

For all individuals with SCI admitted to our department, the authors assessed the comprehensive neurogenic function and general health status after their admission, including the International Standards for Neurological Classification of Spinal Cord Injury (ISNCSCI), complete blood count, electrolyte analysis, urine analysis (a standard urine test including the count of leukocytes and bacteria), and an ultrasound of the upper and lower urinary tracts. After ruling out existing UTI, dilatation of the upper urinary tract, and severe electrolyte disturbances, IC was initiated using a standard protocol recommended in published guidelines (16). IC was performed 4–6 times using disposable catheters per day for regular, complete bladder emptying; in addition, fluid intake was controlled to 125 mL/h and 2,000 mL/day to prevent over-distension of the bladder and to reduce the risk of upper urinary tract damage. Nurses performed *SIC* from January 2015 to December 2017 using iodophor for sterilization before each catheterization because it was required for catheterization to be strictly sterile according to the hospital regulation. From January 2018, CIC was encouraged in the updated version of the local regulation for catheterization (17), and then, it was performed with a clean technique without sterilization, either by SCI patients themselves or by their caregivers. Whether to use bladder irrigation depended on the preference of rehabilitation clinicians rather than a purposeful choice of intervention. Bladder irrigation was used with 500 mL of 0.9% sterile saline bladder infusion at 8 pm every day after the last catheterization before sleep.

### Diagnosis of urinary tract infection

Three criteria were used to define UTI in this study: (1) bacteriuria; (2) pyuria; and (3) signs or symptoms suggestive of UTI (16). Common symptoms and signs suggestive of UTI included fever, cloudy urine, bladder pain, spasticity, malaise, new occurrence or aggravation of urinary incontinence, autonomic dysreflexia, and other symptoms suggestive of UTI. In this study, fever was defined as axillary temperature above 37.2°C. Once a UTI was suspected from symptoms and signs, urine culture and urine analysis were performed using an IC specimen. Bacteriuria was determined as ≥10^2^ colony-forming units (CFU)/mL in an IC specimen culture. Pyuria was determined as white blood cell (WBC) count of ≥30/mL in urine analysis.

In all cases, UTI was diagnosed and treated with antibiotics when signs/symptoms suggestive of UTI and either bacteriuria or pyuria were present. The diagnosis of UTI was made by the attending physician on call based on the criteria above. The use of antibiotics was then determined via consultation with a specialist in infectious disease.

### Data collection

The following data were collected from the electronic medical records: (1) basic information, including demographics, date, etiology, and neurological level of injury (NLI), American Spinal Injury Association Impairment Scale (AIS) grade, history of treated UTIs before started IC, comorbidity of diabetes, and concurrent use of antibiotics for infections other than UTIs; (2) baseline results of urine analysis before performing IC; (3) data related to bladder management, including start date of IC, use of bladder irrigation, method of IC (sterile or clean), and cessation of IC (date and reason of cessation); and (4) data related to UTIs, including date of diagnosis, results of urine analysis, and urine culture.

### Data analysis

The statistical analyses were performed using SPSS version 22.0 (IBM, New York, USA). The level of significance was set at a value of *p* of <0.05. Summary statistics were described by mean and standard deviation (SD) for continuous variables with reasonably symmetric distributions and by median and upper/lower quartiles otherwise.

The occurrence rate of UTI was calculated as the number of UTI events per 100 person-days, and for binominal proportions, the Wilson 95% confidence intervals (CIs) were calculated (18–20).

To explore factors associated with UTI after IC, multivariate logistic regression was used to analyze the significance of the association between these factors and UTI occurrence based on all participants. The study population was stratified by the following independent factors, respectively: sex (male vs. female), age, etiology (traumatic vs. non-traumatic), neurological category (divided as C1-C8 AIS A/B/C, T1-S5 AIS A/B/C, and all AIS D), history of cured UTIs before performing IC (yes or no), comorbidity of diabetes (yes or no), use of antibiotics for infections other than UTIs (yes or no), increased WBC count (≥30/mL) at baseline urine analysis (yes or no), increased bacteria count (≥6,000/mL) at baseline urine analysis (yes or no), method of IC (SCI vs. CIC), and use of bladder irrigation (yes or no). For the regression model, backward stepwise selection was used in order to consider the effects of all variables simultaneously and to eliminate the potential influence of collinearity between variables. The odds ratio and 95% CIs were calculated to reveal the potential association with UTIs for each factor.

## Results

### Characteristics of participants

**Figure 1:**
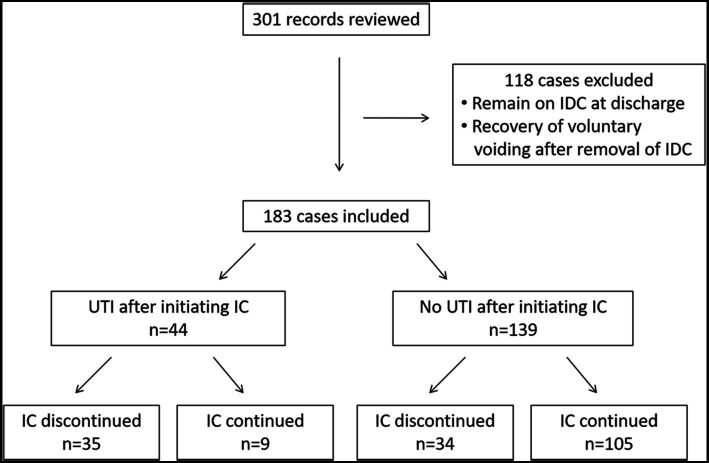
Procedures of data collection. IDC, indwelling urethral catheter; IC, intermittent catheterization; UTI, urinary tract infection.

We reviewed 301 SCI medical records and excluded patients who continued using IDC during inpatient stay or were able to voluntarily void their bladder immediately after the removal of IDC without performing IC. A total of 183 individuals were included in the study (Figure 1). The mean (SD) length of stay was 18.3 (4.3) days. IC was initiated for all 183 participants during their inpatient stay. The median (lower/upper quartiles) days between admission and the start of IC were 6.0 (3.0–8.0) days. The demographic characteristics, neurological category, comorbidities, and medication that may influence the occurrence of UTIs are presented in Table 1. Antibiotics were used in 21 (11.5%) cases for the treatment of pneumonia and/or infectious diarrhea rather than UTIs.

**Table 1:** General characteristics of the study population.

	Total sample (*n* = 183)
Age, median (lower/upper quartiles)	49.0 (37.0–59.0)
Sex	
Male patients, *n* (%)	123 (67.2)
Female patients, *n* (%)	60 (32.8)
Etiology	
Sports, *n* (%)	3 (1.6)
Assault, *n* (%)	12 (6.6)
Motor vehicle accidents, *n* (%)	38 (20.8)
Falls, *n* (%)	60 (32.8)
Other traumatic injury, *n* (%)	12 (6.5)
Non-traumatic injury, *n* (%)	58 (31.7)
Time post-injury in months, median (lower/upper quartiles)	2.0 (1.0–3.0)
Neurological category	
C1-C8, AIS A/B/C, *n* (%)	39 (21.3)
T1-S5, AIS A/B/C, *n* (%)	75 (41.0)
All AIS D, *n* (%)	69 (37.7)
Length of stay (days), mean (SD)	18.3 (4.3)
Diabetes	
No, *n* (%)	152 (83.1)
Yes, *n* (%)	31 (16.9)
History of cured UTIs before initiating IC	
No, *n* (%)	171 (93.4)
Yes, *n* (%)	12 (6.6)
Concurrent use of antibiotics for infections other than UTI	
No, *n* (%)	162 (88.5)
Yes, *n* (%)	21 (11.5)

AIS, American Spinal Injury Association Impairment Scale; IC, intermittent catheterization; SD, standard deviation; UTI, urinary tract infections.

### Summary of data related to bladder management and UTI events

Data related to bladder management and UTI are shown in Table 2. Bladder irrigation was used in 62 (33.9%) participants daily during the inpatient stay without missing any procedure. The most frequently reported reason for the cessation of IC during hospitalization was UTIs, followed by recovery of voluntary voiding. Five (7.2%) participants failed to follow the strict fluid intake schedule due to comorbidities, such as pneumonia, diarrhea, neurogenic shock, and severe hyponatremia that needed fluid supplements for treatment and hence discontinued IC.

**Table 2:** Summary of data related to the laboratory findings and bladder management (*n* = 183).

Laboratory findings and bladder management	
Baseline urine analyses (*n* = 183)	
White blood cell count (per mL), median (lower/upper quartiles)	52 (11–328)
Increased white blood cell count (≥30/mL), *n* (%)	100 (54.6)
Bacteria count (per mL), median (lower/upper quartiles)	331 (16–6,709)
Increased bacteria count (≥6,000/mL), n (%)	47 (25.7)
Days between admission and initiation of IC (*n* = 183), median (lower/upper quartiles)	6.0 (0.-8.0)
Use of bladder irrigation (*n* = 183)	
No, *n* (%)	62 (33.9)
Yes, *n* (%)	121 (66.1)
Method of IC (*n* = 183)	
*SIC*, *n* (%)	127 (69.4)
CIC, *n* (%)	56 (30.6)
Cessation of IC (*n* = 183)	
No, *n* (%)	114 (62.3)
Yes, *n* (%)	69 (37.7)
Reasons for cessation of IC (*n* = 69)	
UTI, *n* (%)	35 (50.7)
Recovery of voluntary voiding, *n* (%)	18 (26.2)
Pain/bleeding/stenosis of the urethra, *n* (%)	6 (8.7)
Failure to follow the fluid intake schedule due to comorbidities, *n* (%)	5 (7.2)
Resistance/poor compliance to the IC intervention, *n* (%)	4 (5.8)
Dilatation of the upper urinary tract/hydronephrosis, *n* (%)	1 (1.4)
UTI events (*n* = 183)	
No, *n* (%)	139 (76.0)
Yes, *n* (%)	44 (24.0)
Days between IC initiation and UTI (*n* = 44), median (lower/upper quartiles)	4.0 (1.6–8.0)

IC, intermittent catheterization; SD, standard deviation; UTI, urinary tract infections.

UTI events occurred in 44 (24.0%) participants in this study. Median (lower/upper quartiles) days between IC initiation and UTI was 4.0 (1.6–8.0) days; 35 out of the 44 (79.5%) participants with UTI reversed to IDC. For the other 9 (20.5%) participants, IC was continued along with oral or intravenous treatment of antibiotics. All 44 participants with UTIs were treated with quinolones or cephalosporins for 5–14 days based on the antibiotic susceptibility test.

### Multivariate logistic regression of the potential factors related to the occurrence of UTI

No collinearity between the variables was found, with all the variance inflation factors between 1.0 and 1.4. The results of logistic regression for several factors and UTI after performing IC are listed in Table 3. The use of antibiotics for infections other than UTI was found to be associated with a reduction in UTIs after performing IC, with an odds ratio of 0.20 (95% CI, 0.04–0.99, *p* = 0.022). Female sex and use of bladder irrigation were also associated with a lower occurrence rate of UTIs after performing IC, with an odds ratio of 0.38 (95% CI, 0.16–0.89, *p* = 0.019) and 0.24 (95% CI, 0.11–0.54, *p* < 0.001), respectively. Age, etiology of injury, neurological category, history of cured UTIs before performing IC, comorbid diabetes, increased WBC count at baseline urine analysis, increased bacteria count at baseline urine analysis, and method of IC were not significantly associated with UTI in the early phase after performing IC.

**Table 3:** Summary of multivariate logistic regression and odds ratio of UTIs.

	Odds ratio (95% CI)	*P*
Sex		0.019
Male patients	Ref	
Female patients	0.38 (0.16–0.89)	
Age	0.99 (0.96–1.02)	0.571
Etiology		0.467
Traumatic	Ref	
Non-traumatic	0.72 (0.30–1.75)	
Neurological category		0.104
C1-C8, AIS A/B/C	Ref	
T1-S5, AIS A/B/C	2.66 (0.99–7.18)	
All AIS D	1.04 (0.42–2.56)	
History of cured UTIs before IC		0.605
No	Ref	
Yes	1.51 (0.33–6.91)	
Diabetes		0.089
No	Ref	
Yes	2.32 (0.89–6.05)	
Use of antibiotics for infections other than UTI		0.022
No	Ref	
Yes	0.20 (0.04–0.99)	
Increased white blood cell count at baseline urine routine test		0.189
No	Ref	
Yes	1.70 (0.76–3.76)	
Increased bacteria count at baseline urine routine test		0.753
No	Ref	
Yes	1.17 (0.44–3.13)	
Method of IC		0.065
*SIC*	Ref	
CIC	0.44 (0.18–1.08)	
Use of bladder irrigation		<0.001
No	Ref	
Yes	0.24 (0.11–0.54)	

AIS, American Spinal Injury Association Impairment Scale; CI, confidence interval; IC, intermittent catheterization; CIC, clean IC; Ref, reference category; SIC, sterile IC; UTI, urinary tract infections.

### Occurrence rate of UTI

The overall occurrence rate of UTI after performing IC in the 183 participants was 1.31 (95% CIs, 0.96–1.77) events per 100 person-days during the inpatient stay. The occurrence rate of UTI in female participants was 0.65 (95% CIs, 0.29–1.39) events per 100 person-days; the occurrence rate of participants using antibiotics for infections other than UTI was 0.50 (95% CIs, 0.09–1.91) events per 100 person-days; and the occurrence rate of participants receiving bladder irrigation was 0.92 (95% CIs, 0.58–1.44) events per 100 person-days.

## Discussion

IC is widely accepted as the gold standard for bladder management in appropriate individuals with SCI. Our study suggests that UTI in the early phase after performing IC is common in individuals with acute SCI and is associated with cessation in the early phase after the start of IC. Female patients, patients who took antibiotics treatment for other infections, and those who were receiving bladder irrigation were associated with a lower risk of UTI when switching from IDC to IC.

The overall occurrence rate and 95% CIs of UTI after performing IC in our sample was 1.31 (0.96–1.77) events per 100 person-days, which was higher than the results reported in previous studies, as summarized in Table 4 (3, 7, 14, 19, 20, 22, 23). The UTI definition used in most of these studies was a positive bacterial culture and the presence of a new sign or symptom that is suggestive of antibiotic treatment. The occurrence rate of UTIs in the SCI population has been reported as 0.55–1.1 episodes per 100 person-days (3, 19, 20). Specifically, the occurrence rate of UTIs in individuals performing CIC varies, ranging from 0.22 to 0.96 per 100 person-days (recalculated from the original description of 0.8–3.5 per year) (14). The higher occurrence rate of UTI in our study may be attributed to a relatively shorter length of stay for the participants included. Although data regarding the length of stay were not clearly described in all the previous studies, the reported average (median) length of stay/observation was more than 3 months (3, 19, 20, 23). In contrast, the mean length of stay in our study was 18.4 days, and patients were discharged to specialized rehabilitation centers due to the regulations for admission and discharge from the hospital. As mentioned in the results, no recurrent UTI was found at discharge in this study, which may partly be due to the relatively short time of stay.

**Table 4:** Overview of the IR of UTI in the published studies.

Year/Author/Country	Study design	Sample size	Population	Average/median length of stay/observation	IR of UTI
1995, Bennett, USA (21)	Prospective	54	SCI patients undergoing IC	6 weeks	One infection in every 146 self- or staff-intermittent catheterizations; Significantly more infection events in female patients
2000, Esclarín, Spain (3)	Prospectively cohort study	128	Acute SCI	207 days	0.68 episode per 100 person-days in the study population; 0.41episode per 100 person-days in individuals using CIC; Patient sex not associated with an increased risk of UTI
2008, Woodbury, Canada (22)	National survey	912	Traumatic and non-traumatic SCI	–	2.6 ± 2.6 UTIs in the past 12 months in IC users; Female IC users had a significantly greater number of UTIs than male IC users
2015, Krassioukov, Canada (23)	Questionnaire-based survey	61	Paralympic wheelchair athletes with traumatic SCI (>1 year after injury), using CIC	–	Individuals reusing catheters: 4 ± 3 (Means ± SD) UTIs per year; Individuals never reusing catheters: 1 ± 1 UTI per year
2016, Krebs, Switzerland (24)	Retrospective	1,104	Chronic (>12 months) traumatic or non-traumatic SCI	–	The occurrence rate of UTI: 70.5% (95% CIs: 65.9–74.8%) per year in individuals using IC; Patient sex not associated with risk of UTI
2019, Anderson, Switzerland (20)	Multicenter, prospective cohort study	369	Newly diagnosed with a traumatic or non-traumatic SCI	122 days	0.55 per 100 person-days (95% CIs: 0.49–0.62) in the study population; 0.68 (95% CI: 0.52–0.90) per 100 person-days in individuals using assisted IC; 0.53 (95% CIs: 0.39–0.71) per 100 person-days in individuals using self-IC; Female/male IR ratio of UTI: 0.76
2019, Derek, Australia (7)	Prospectively cohort study	143	Acute SCI	104 days	6.84 per 1,000 person-days in individuals performing CIC; Significantly more UTIs in male patients (data not shown)
2019, Kennelly, USA (14)	Review	–	Community-based population (>12 months after injury)	–	Ranged from 0.8 to 3.5 per year
2020, Goodes, Australia (19)	Retrospective	70	Acute traumatic SCI	97.6 days	1.1 starts per 100 person-days in the study population; 0.8–2.0 starts per 100 person-days in individuals using staff-IC or self-IC; Female/male odds ratio of UTI: 6.1

CI, confidence interval; IC, intermittent catheterization; CIC, clean IC; IR, incidence rate; SCI, spinal cord injury; UTI, urinary tract infections.

The influence of sex factors on the UTI risk among people with SCI remains inconclusive (summarized in Table 4). In our study, female patients were associated with a lower occurrence rate of UTIs in the early phase after performing IC, with an odds ratio of 0.38. Similar results were reported in a population-based study from Switzerland that female sex was associated with fewer UTIs (20). In new SCI patients in Australia, UTI risk was high in male patients (7). However, the sex factor was not associated with UTI risk after SCI in a prospective study in Spain (3) and in another study among people with chronic neurogenic lower urinary tract dysfunction (24). Three more studies found UTIs were more common in female SCI patients with or without performing IC (19, 21, 22), but these studies were based on the numbers of UTI events rather than calculated IR. Whether the IR of UTI was different between the male and the female patients remains unclear and may be of interest for further research.

It is not surprising that the use of antibiotics for infections other than UTIs could reduce the occurrence rate of UTIs. The use of broad-spectrum antibiotics for other infections might also eliminate or inhibit the bacteria species in urine. Recently, a randomized, open-label trial has been published to use continuous low-dose antibiotic prophylaxis for adults who use CIC with repeated UTIs (25). It was suggested that continuous antibiotic prophylaxis was effective in reducing UTI frequency in IC users, but the potentially increased risk of drug resistance was a concern. A weekly oral cycling antibiotics strategy was demonstrated to be safe and effective for the prevention of UTIs in the SCI population (26). As to the SCI patients in the early phase after switching to IC, prophylactic use of antibiotics still needs further investigation to evaluate the balance between effect and risk of developing antibiotic resistance.

Among the three factors found to reduce the risk of UTI after performing IC, bladder irrigation was more manageable. Although there is weak evidence supporting intermittent bladder irrigation with antibiotics (27), no advantage was detected for antibiotics over saline in reducing the bacterial load and inflammation in urine (28). Moreover, the effect of bladder irrigation on the degree of bacteriuria and pyuria was not significant (28), which implied that the relationship between bladder irrigation and reduced risk of UTIs remains controversial. Further research with well-designed clinical trials is necessary to investigate the potential effect of bladder irrigation.

It is an important fact that the participants in this study were on IDC before performing IC. As chronic bacteriuria is common in individuals on IDC, the potential influence of “concealed” UTIs at baseline was taken into account. Patients with existing UTI (i.e., evidence of bacteriuria/pyuria and signs/symptoms suggestive of UTI) were ruled out for IC and were naturally excluded from this study. For the participants included, baseline results of urine analysis before performing IC were collected. In order to explore the potential influence of asymptomatic bacteriuria at baseline on UTI occurrence in the early phase after performing IC, “history of cured UTIs before performing IC,” “increased WBC count (≥30/mL) at baseline urine analysis,” and “increased bacteria count (≥6,000/mL) at baseline urine analysis” were considered as three variables in multivariate logistic regression. As presented in Table 3, none of them showed a statistically significant association with UTI occurrence after performing IC.

This study has several limitations. The study design is retrospective in nature, making it difficult to collect adequate data for the comparison before and after performing IC. For example, important information regarding urodynamics, hand function and urine analysis after performing IC were absent in most of the participants without UTIs, making it impossible to investigate the influence of these factors on UTI occurrence. Besides, the recurrence of UTI after performing IC was not observed due to the short length of stay of this study. Further follow-up research is needed to explore the long-term effect of the various factors that may affect the occurrence of UTIs after performing IC. Third, although the use of antibiotics and bladder irrigation were related to a lower risk of UTI after performing IC, the influence of type and duration of antibiotics treatment as well as the duration of bladder irrigation were unknown due to the limited data collected retrospectively. Prospective research is needed to evaluate these factors and to explore other modifiable factors on the occurrence of UTI after performing IC in SCI individuals.

## Conclusion

Based on our study results, UTI in the early phase after performing IC is prevalent in patients with acute SCI. Female sex, treatment of antibiotics for other infections, and use of bladder irrigation might be associated with a lower occurrence rate of UTI when IC was initiated. Antibiotic prophylaxis and routine bladder irrigation might be helpful to reduce the occurrence of UTI for SCI individuals when transitioning from IDC to IC, but further research is needed to provide more evidence on potential risk factors and their substantial influence on UTI occurrence in the early phase after performing IC.

## Data availability statement

The raw data supporting the conclusions of this article will be made available by the authors, without undue reservation.

## Ethics statement

The studies involving humans were approved by the Clinical Research Ethics Board of Peking University Third Hospital. The studies were conducted in accordance with the local legislation and institutional requirements. Written informed consent for participation was not required from the participants or the participants’ legal guardians/next of kin in accordance with the national legislation and institutional requirements.

## Author contributions

HX: Writing – original draft, Writing – review & editing, Investigation, Methodology. HD: Investigation, Writing – review & editing. BL: Methodology, Validation, Writing – review & editing. XY: Validation, Writing – review & editing. XL: Methodology, Supervision, Writing – review & editing. GC: Supervision, Writing – review & editing. NL: Investigation, Supervision, Writing – original draft, Writing – review & editing, Methodology. FB-S: Methodology, Supervision, Validation, Writing – review & editing.
